# Non-pressurized percutaneous endoscopic transforaminal lumbar discectomy in the treatment of cauda equina syndrome caused by lumbar disc herniation

**DOI:** 10.3389/fsurg.2025.1674943

**Published:** 2025-12-04

**Authors:** Shangjv Gao, Lifang Shi, Can Cao, Jingchao Wei, Wenjie Lv, Wenyi Li

**Affiliations:** Department of Orthopedics, Hebei General Hospital, Shijiazhuang, Hebei, China

**Keywords:** transforaminal endoscopic surgery, surgical technique, lumbar disc herniation, technical modification, cauda equina syndrome

## Abstract

**Objective:**

Cauda equina syndrome (CES) is a rare but devastating condition in spine surgery. There are various surgical decompression methods, but the clinical outcomes are not always satisfactory. We introduce the surgical technique of non-pressurized percutaneous endoscopic transforaminal lumbar discectomy (PETD) technique in the treatment of cauda equina syndrome caused by lumbar disc herniation, and to evaluate its clinical efficacy compared to traditional transforaminal lumbar interbody fusion (TLIF).

**Methods:**

A retrospective study was conducted on 51 cases diagnosed with lumbar disc herniation complicated by cauda equina syndrome. They were divided into two groups, 23 cases in the PETD group and 28 cases in the TLIF group. The surgical procedure and technical details of non-pressurized PETD technique were described. Visual analogue scale (VAS) for low back pain and leg pain, Japanese Orthopaedic Association scores (JOA), Oswestry Disability Index (ODI), and the cauda scale (TCS) were used to evaluate the clinical efficacy between the two groups.

**Results:**

There were no significant differences in baseline data between the two groups. Significant improvements were observed at the final follow-up in both PETD and TLIF groups. The VAS for low back pain and ODI at discharge were lower in the PETD group than those in the TLIF group (both *P* < 0.05). There were no significant differences in low back pain VAS, leg pain VAS, JOA score, ODI score, and TCS score between the two groups at preoperative and final follow-up. The complications included two cases of intraoperative neck pain and one case of recurrent disc herniation in the PETD group, and one case showed adjacent segment degeneration at the final follow-up in the TLIF group.

**Conclusion:**

In this preliminary retrospective study, the non-pressurized PETD technique was associated with similar medium-term outcomes and a faster short-term recovery compared to TLIF. These findings require validation in larger, prospective, randomized controlled trials to establish comparative efficacy.

## 1 Introduction

Cauda equina syndrome (CES) is a rare but devastating condition, which can result in a series of catastrophic consequences, including urinary incontinence, faecal incontinence, loss of sexual function, saddle anaesthesia, neuropathic pain, and sometimes paralysis of the legs. This devastating condition arises from compression of the cauda equina nerve, due to cauda equina nerve as terminal nerve bundle that serves to supply the lower extremities, perineum, perianal region, and the bladder (1). Therefore, lumbar disc herniations (LDH) are the leading cause of CES, representing approximately 45% of the total CES cases (2). Since these catastrophic consequences severely affect the daily lives of patients, the treatment and prognosis are particularly critical.

Urgent surgical decompression is the only effective treatment for CES (3). It is widely believed that the clinical efficacy is highly correlated with the degree and duration of cauda equina nerve compression, with symptoms persisting for more than 48 h resulting in irreversible consequences for the nerves (4). There are various surgical decompression methods, but the clinical outcomes are not always satisfactory. Even years after surgery, two-thirds of patients still experience residual bladder or sexual dysfunction (5, 6). The optimal surgical approach remains controversial, with open surgery appearing to be more commonly used and minimally invasive surgery becoming increasingly popular (7).

Transforaminal lumbar interbody fusion (TLIF), as a classic open surgical procedure, has the advantages of providing aggressive, wide decompression of the neural elements and restoration of spinal stability, but it is also associated with potentially higher risk for complications such as post-surgical pain, potential for segmental destabilization, infections, and a longer recovery period (8–10). Percutaneous endoscopic transforaminal lumbar discectomy (PETD), characterized as minimally invasive, offers advantages such as reduced approach related morbidity and possibly quicker recovery time. However, there is concern that PETD does not allow for an adequate wide decompression of the neural elements, due to the minimal access corridor (11–13). However, the potential risk of iatrogenic pressure increases on the compromised cauda equina during traditional PETD, and nerve damage itself is a complication of spinal endoscopic surgery with an incidence rate of 0.4%–1.0% (14, 15). Moreover, PETD and its comparative efficacy against gold-standard fusion techniques, remains poorly understood.

In this study, we introduced the non-pressurized PETD technique in detail. As a modified PETD technique, the highlight of this technique is that during the process of removing the herniated disc to decompress the nerve roots and cauda equina nerves, no traction or pushing of the nerves was performed, and the intradiscal pressure did not increase, thereby avoiding the risk of iatrogenic cauda equina nerve injury. Further, we evaluated its clinical efficacy of LDH complicated by CES, compared to traditional TLIF.

## 2 Materials and methods

### 2.1 Study population

This study was a retrospective study approved by the hospital ethics committee (No. 2024-LW-093). Cases undergoing surgical treatment because of LDH complicated by CES were included from January 2016 to December 2020 at Hebei General Hospital.

The clinical diagnosis of CES is widely accepted based on dysfunction of the cauda equina nerve. In this study, CES must present one or more of the following symptoms or signs: (1) bladder and/or bowel dysfunction, (2) reduced sensation in the saddle area, and (3) sexual dysfunction. For CES secondary to LDH, the symptoms are usually accompanied by back and leg pain, and lower limb motor or sensory changes, but these are not essential to the diagnosis of CES (16). And clinical stage was evaluated according to Shi's classification (17).

#### 2.1.1 Inclusion and exclusion criteria

Inclusion criteria consist of (1) clinical manifestations of perineal sensory disturbance, decreased anal sphincter strength, bladder dysfunction, and/or sexual dysfunction; (2) CT or MRI imaging findings of severe compression of cauda equina nerves due to single-level disc herniation; (3) surgical treatment by non-pressurized PETD or traditional TLIF; (4) complete clinical data with a follow-up period of ≥1 year.

Exclusion criteria include (1) disc herniation at L5-S1 level with high iliac crest, which is difficult to decompress by transforaminal PETD; (2) nerve compression due to severe ligamentum flavum hypertrophy, facet joint hypertrophy or other signs; (3) imaging findings observed such as lumbar instability, lumbar spondylolisthesis, spinal deformity or neural malformation on imaging; (4) cauda equina compression caused by fractures, infections or spinal tumors; (5) previous lumbar surgery.

#### 2.1.2 General information

After screening, a total of 51 cases were involved in this study, including 22 males and 29 females, with mean age of 38.6 ± 13.3 years (range 16–69 years). The time from the onset of CES symptoms to surgery was 6.7 ± 3.9 days (range 1–17 days). There were 21 cases in the early stage, 26 cases in the middle stage and 4 cases in the late stage, and the preclinical stage was not included in the study. All patients underwent surgery within 48 h of hospitalization, including 1 case at L1–2, 6 cases at L2–3, 15 cases at L3–4, 22 cases at L4–5, and 7 cases at L5-S1 (Table 1).

**Table 1:** Demographic data between PETD group and TLIF group.

Variables	PETD group	TLIF group	$x^{2}$/*t*	*P* value
*n*	23	28		
Age (years, x ± s)	34.9 ± 8.6	41.6 ± 15.8	−1.82	0.075
Gender			0.27	0.601
Male	9 (39.13)	13 (46.43)		
Female	14 (60.87)	15 (53.57)		
Duration of symptoms (days, x ± s)	7.7 ± 4.6	5.9 ± 3.1	1.59	0.118
Clinical stage			NA*	0.913
Early	10 (43.48)	11 (39.29)		
Middle	11 (47.83)	15 (53.57)		
Late	2 (8.69)	2 (7.14)		
Surgical segment (cases)			NA*	0.860
L1/2	0 (0.00)	1 (3.57)		
L2/3	2 (8.69)	4 (14.29)		
L3/4	6 (26.09)	9 (32.14)		
L4/5	11 (47.83)	11 (39.29)		
L5/S1	4 (17.39)	3 (10.71)		

* Fisher's exact test was used to calculate the *P* value directly, obviating the reporting of a chi-square statistic.

All patients were informed of the surgical plan and risks of PETD and TLIF before surgery, and the surgical method was chosen by the patients and their families after consultation. Based on the surgical methods, 23 cases who underwent non-pressurized PETD surgery were considered as the PETD group, while 28 cases who underwent traditional TLIF surgery were considered as the TLIF group. There were no significant differences between the two groups in terms of gender, age, duration of symptoms before surgery, clinical stage, or surgical segment (all *P* > 0.05), indicating comparability (Table 1).

### 2.2 Non-pressurized PETD surgical procedure

On the basis of the traditional PETD (18), the non-pressurized PETD surgical procedure modifies the operational details involved in the process of advancing the working channel to the target intervertebral foramen. In the traditional PETD, the working channel was placed directly into the target intervertebral foramen before decompression, and the insertion of the working channel into the spinal canal space increased the intraspinal pressure. In contrast to this traditional step, the key point of the “non-pressurization” technique is to perform decompression of the target disc before insertion of the working channel, while the working channel was advanced gradually into the intervertebral foramen concomitantly with the decompression. This technique improvement avoids compression, traction, and increased intraspinal pressure, so we named it “non-pressurized technique”. The non-pressurized PETD surgical procedure was introduced step by step as follows, and the precautions, possible problems and solutions were provided at each step.

#### 2.2.1 Preoperative preparation

Prior to surgery, lumbar spine CT, MRI, and anteroposterior and lateral flexion-extension x-rays were conducted. If patients presented with bladder dysfunction, residual urine measurement was performed. Patients were guided by medical staff to practice adapting to the surgical position. The surgical position was prone with chest and iliac cushioning, hips flexed approximately 30°, and knee joints flexed approximately 15°. This position ensured patient comfort, sustained duration, and alleviated neurological symptoms to some extent.

#### 2.2.2 Anesthesia method

Surgery was performed under local anesthesia combined with conscious sedation analgesia. Local anesthetics consisted of 30 mL of 0.5% lidocaine and 0.25% ropivacaine for infiltration anesthesia around the puncture track and target intervertebral foramen. Sedation analgesics were administered intravenously at a rate of 0.1–0.5*μ*g/kg·h using a microinfusion pump with a concentration of 4μg/mL of dexmedetomidine. Throughout the procedure, continuous monitoring of electrocardiogram, blood pressure, respiration, and pulse oximetry was conducted under the supervision of an anesthesiologist.

#### 2.2.3 Establishment of working channel

Under fluoroscopic guidance from a C-arm x-ray machine, the surface marking of the puncture path was conducted. The head tilt angle of the puncture path was approximately 10°-15°, and the distance from the skin puncture point to the spinous process midline was 11–13 cm, adjusted according to the patient's body shape. After local anesthesia of the skin, lumbar fascia, and paraspinal muscles, puncture was performed under C-arm x-ray guidance. The target point of the puncture was the lateral recess of the surgical segment, with the needle tip positioned at the inner edge of the upper and lower articular processes on anteroposterior view and at the posterior edge of the target intervertebral space on lateral view.

After puncture, local anesthetic was slowly injected through the needle to perform infiltration anesthesia on the intervertebral foramen and surrounding tissues. It should be noted that local anesthetic is about 10 mL in volume, and larger volumes may enter the spinal canal through the intervertebral foramen, leading to increased intraspinal pressure and subsequent exacerbation of nerve compression. Additionally, fast injection rates can also increase this risk. Subsequently, the puncture needle was withdrawn to stabilize it on the lateral aspect of the upper articular process, the outer area of the intervertebral foramen. The needle tip was positioned at the upper articular process under lateral fluoroscopy, and at the outer edge of the pedicle under anteroposterior fluoroscopy.

**Figure 1:**
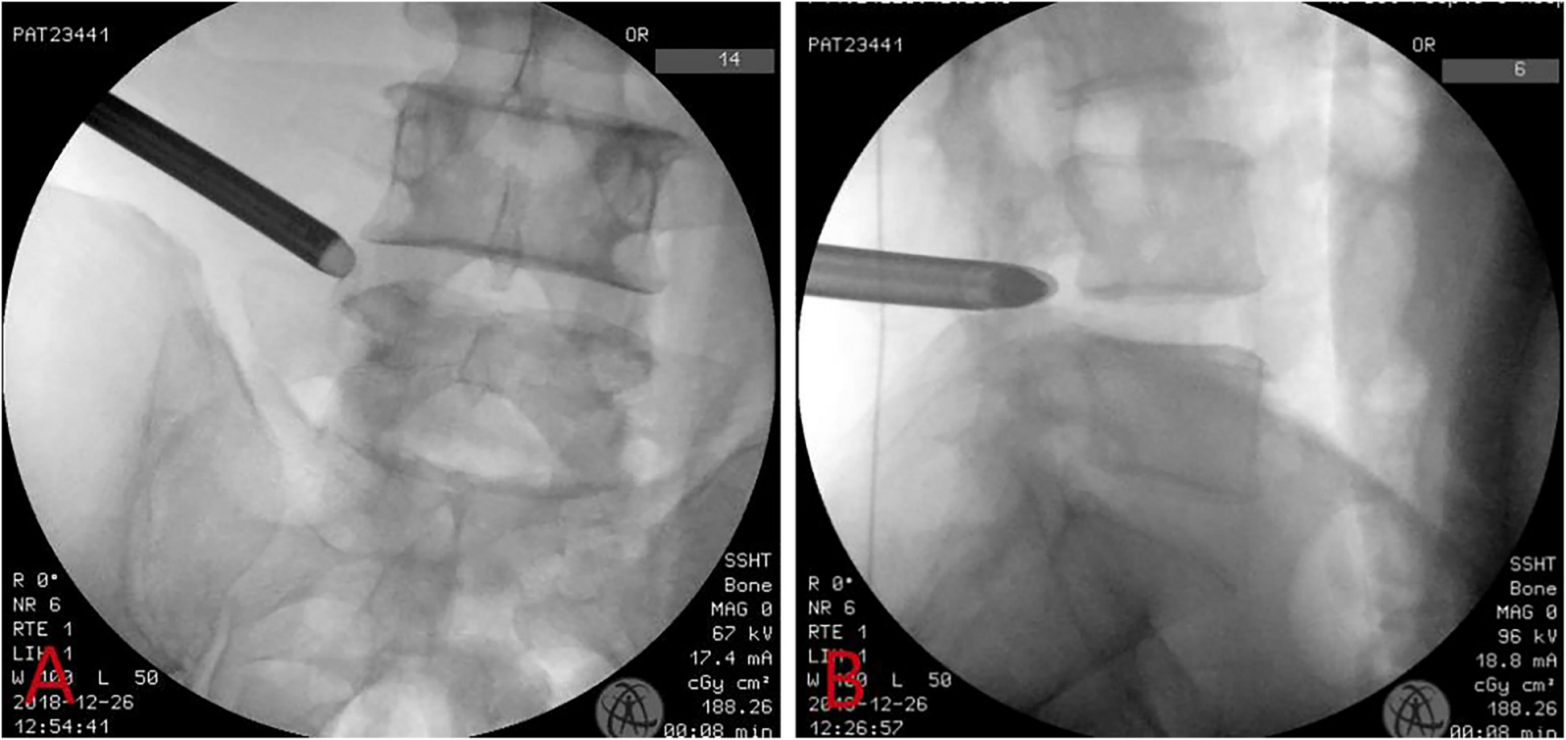
Intraoperative fluoroscopic images showing the working channel was located at the outer edge of the upper and lower vertebral pedicles in the anteroposterior view **(A)** and the intervertebral foramen in the lateral view **(B)**.

The puncture needle was then removed, and a guide wire was inserted along with the soft tissue dilator and working channel, which was now positioned at the outer opening of the target segment's intervertebral foramen. Confirmation under fluoroscopy showed that the working channel was located at the outer edge of the pedicle on anteroposterior view (Figure 1A) and in the area of the intervertebral foramen on lateral view (Figure 1B).

#### 2.2.4 Non-pressurized endoscopic procedures

**Figure 2:**
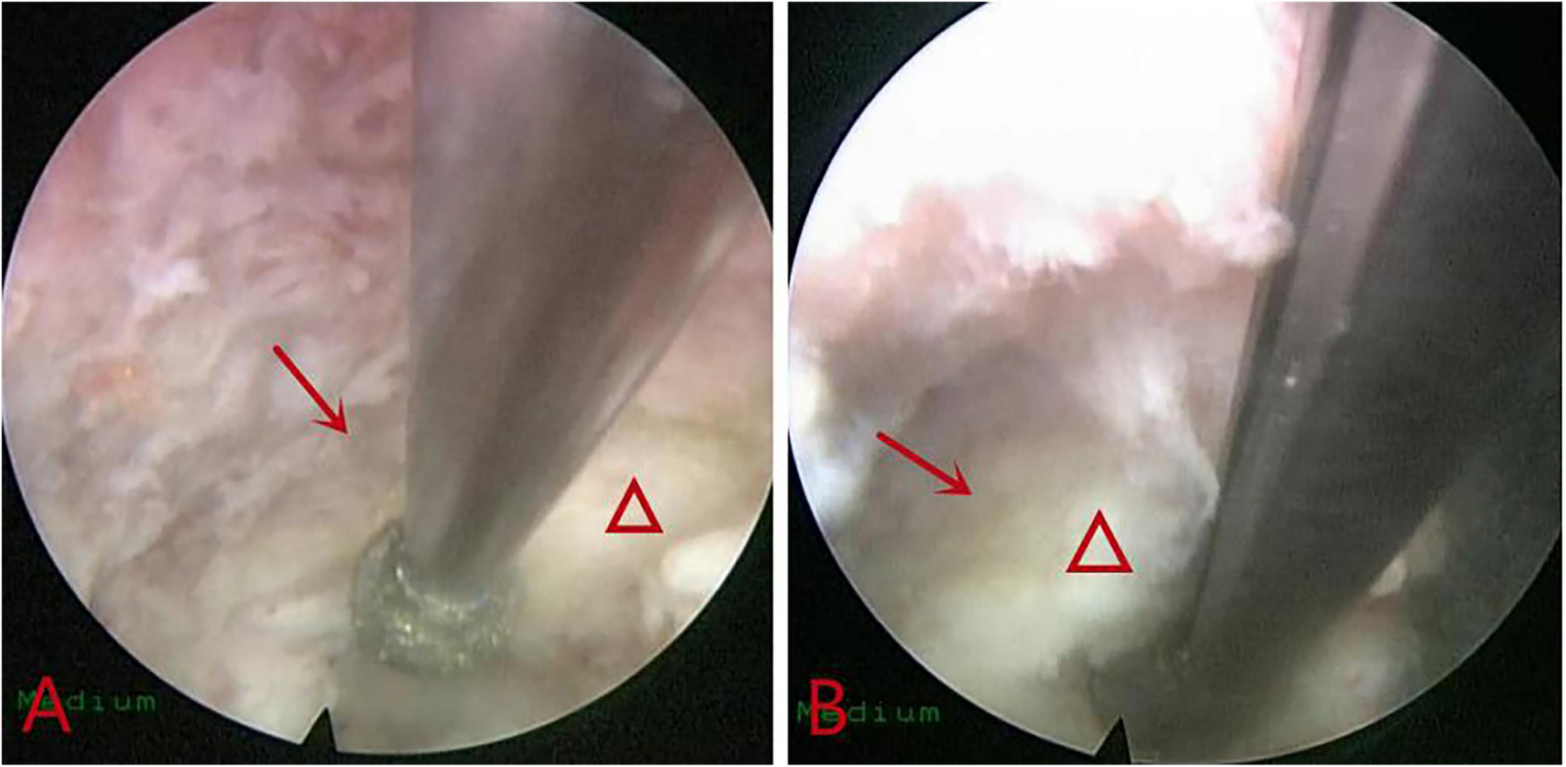
Endoscopic view demonstrating foraminoplasty using a burr **(A)** and Kerrison rongeur **(B)**.

Initially, using a burr (Figure 2A) or Kerrison rongeurs (Figure 2B), the intervertebral foramen was expanded and shaped under direct vision. After widening the area of the intervertebral foramen, the working channel and endoscope were further advanced towards the spinal canal. Adequate dilation of the intervertebral foramen is necessary before pushing the working channel inward, and it should not be forced to avoid increased intraspinal pressure and subsequent nerve damage. During advancement, the patient was continuously assessed for exacerbation of lower limb pain or symptoms related to cauda equina.

When the working channel reached the inner opening of the intervertebral foramen and lateral recess, if extruded nucleus pulposus tissue was visible, it was removed to decompress the spinal canal. However, in cases of secondary CES caused by LDH, which is often central in nature, the herniated disc is obscured by nerve roots and the dura mater, making it invisible in the lateral recess area. In such cases, the endoscope's view was directed towards the ventral aspect of the spinal canal, exposing the ventral aspect of the intervertebral disc and vertebral body. The annulus fibrosus was sharply incised using a basket forceps, and nucleus pulposus inside the disc was removed, achieving intradiscal decompression. Once sufficient space was created within the intervertebral disc, the endoscope was inserted into the disc space, with the herniated disc and nerve located at the 12 o'clock position in the field of view. Due to the endoscope's inherent 30-degree field of view, the herniated disc could be easily observed. Flexible pituitary rongeurs or other bendable instruments were used to separate and remove the intervertebral disc. In some cases, the herniated disc is accompanied by calcification, and the technical details for resecting a calcified disc are described comprehensively in our previous Technical Notes (19). Briefly, the soft portion of the calcified disc was first removed, followed by drilling down the base of the hardened shell portion to allow it to be collapsed into the intervertebral space and removed piece by piece. After disc removal, the posterior longitudinal ligament was visible, and whether to preserve it depended on the pulsation of the dura mater; the primary goal of surgery was thorough decompression rather than preserving the posterior longitudinal ligament.

Finally, radiofrequency ablation of the intervertebral disc was performed. Confirmation of complete disc removal and adequate space for the cauda equina and nerve roots was followed by the completion of the surgery, and the skin was sutured. Effective communication with the patient throughout the surgical procedure was crucial for ensuring safety based on feedback regarding the patient's symptoms.

#### 2.2.5 Postoperative management

After surgery, routine antibiotic prophylaxis was administered for 24 h. Patients remained in bed for 24 h, with axial rotation avoided to prevent epidural hematoma. After 24 h, patients were assisted to get out of bed by turning to the side while wearing a lumbar brace, avoiding bending and lateral rotation. Rest was emphasized within 6 weeks, and strenuous physical activity and sports were avoided for 3 months.

### 2.3 Traditional TLIF surgical procedure

Traditional TLIF is regarded as the classic surgical treatment for LDH (20). The surgical procedure is described as follows.

After successful endotracheal intubation under general anesthesia, patients were placed in a prone position, and the surgical segment was marked on the skin. Standard iodine and alcohol disinfection were performed, followed by draping. A posterior midline incision approximately 6 cm long was made around the surgical segment, and the paraspinal muscles were dissected along the spinous process to expose the lamina and bilateral facet joints. Pedicle screws were placed routinely into the upper and lower pedicles of the fusion segment, and a rod was connected on one side.

A portion of the spinous process of the upper vertebral body of the responsible segment was removed using Kerrison rongeurs and standard double-joint rongeurs to perform a hemi- or total laminectomy. Further resection of the upper and lower facet joints of the upper and lower vertebral bodies was then performed to expose the Kambin triangle. Using an No. 11 blade, the annulus fibrosus was incised, and the intervertebral disc was removed using nucleus pulposus forceps within the intervertebral space. The nerve roots and dura mater were gently pulled towards the midline of the spinal canal to expose the ventral aspect of the nerve roots and dura mater, where the intervertebral disc was located. Intradiscal disc removal and exploration of the nerve were performed to achieve thorough decompression of the nerve roots and cauda equina. Cartilaginous endplates were removed using a chisel and scraper, and the bone bed was adequately prepared. Autogenous bone grafts were placed and compacted, followed by insertion of an appropriately sized interbody fusion cage. Depending on the extent of decompression and the position of the disc herniation, the contralateral facet joints were preserved to enhance spinal stability. If the disc herniation was large or severe disc calcification was present, both facet joints were removed as needed, and decompression was alternately performed on the ventral aspect of the spinal canal from both sides.

After meticulous hemostasis, the wound was closed layer by layer, leaving one drain in place. Routine intravenous antibiotics were administered for 24–48 h postoperatively. If drainage was less than 50 mL over 48–72 h, the drain was removed. Patients were mobilized with lumbar protection, and the lumbar brace was worn for 4–6 weeks.

### 2.4 Evaluation of therapeutic effectiveness

The surgical duration was defined as the time from puncture to closure for non-pressurized PETD, and the time from incision to closure for TLIF. Before discharge, lumbar MRI was performed in the PETD group to evaluate the extent of decompression, while lumbar x-ray was performed in the TLIF group to evaluate the position of internal fixation.

Low back pain and leg pain were assessed using the Visual Analogue Scale (VAS), defined as the score at the time of maximum pain after admission. Neurological dysfunction was assessed using the Japanese Orthopaedic Association (JOA) score, Oswestry disability index (ODI), and the cauda scale (TCS). The TCS is a quantitative scoring system for evaluating cauda equina function (Table 2) (21), which was used to specifically evaluate cauda equina function at three time points: preoperative, discharge, and final follow-up.

**Table 2:** The cauda scale.

Cauda equina function	Score
Perianal Sensation	
No complaints of perianal sensation abnormalities, normal examination	3
Complaints of perianal sensation abnormalities, decreased sensation on examination	2
Markedly decreased perianal sensation (unilateral/bilateral)	1
Absence of perianal sensation (unilateral/bilateral)	0
Anal sphincter muscle strength	
Normal	2
Decreased	1
Absent	0
Bladder function	
Normal	4
Urgency or waiting for urination, but sensation and control are normal	3
Decreased bladder or urethral sensation, but urination control is normal	2
Urinary retention, but no urinary incontinence	1
Painless urinary retention (>500 mL), or urinary incontinence	0

## 3 Statistical analysis

The statistical analysis was conducted using IBM SPSS Statistics (version 21.0; IBM Corp., Armonk, N.Y., USA). Continuous variables such as age, duration from CES symptoms to surgery, surgical duration, length of hospital stay, and follow-up duration were expressed as mean and standard deviation. Gender, clinical stages and surgical segments were represented by frequencies and percentage. Continuous variables including VAS, JOA, ODI and TCS were represented by mean and standard deviation if they followed a normal distribution, and by median and interquartile range if they did not. When the variances of VAS, JOA, ODI, and TCS between groups or before and after surgery were equal, a *t*-test was used for comparison; if the variances were unequal, the Mann–Whitney *U* test was employed. Categorical data were compared using the chi-square test or Fisher's exact test. A significance level of *P* < 0.05 was considered statistically significant. *post hoc* power analysis was performed using PASS software (version 15; NCSS LLC., Kaysville, U.T., USA).

## 4 Result

**Figure 3:**
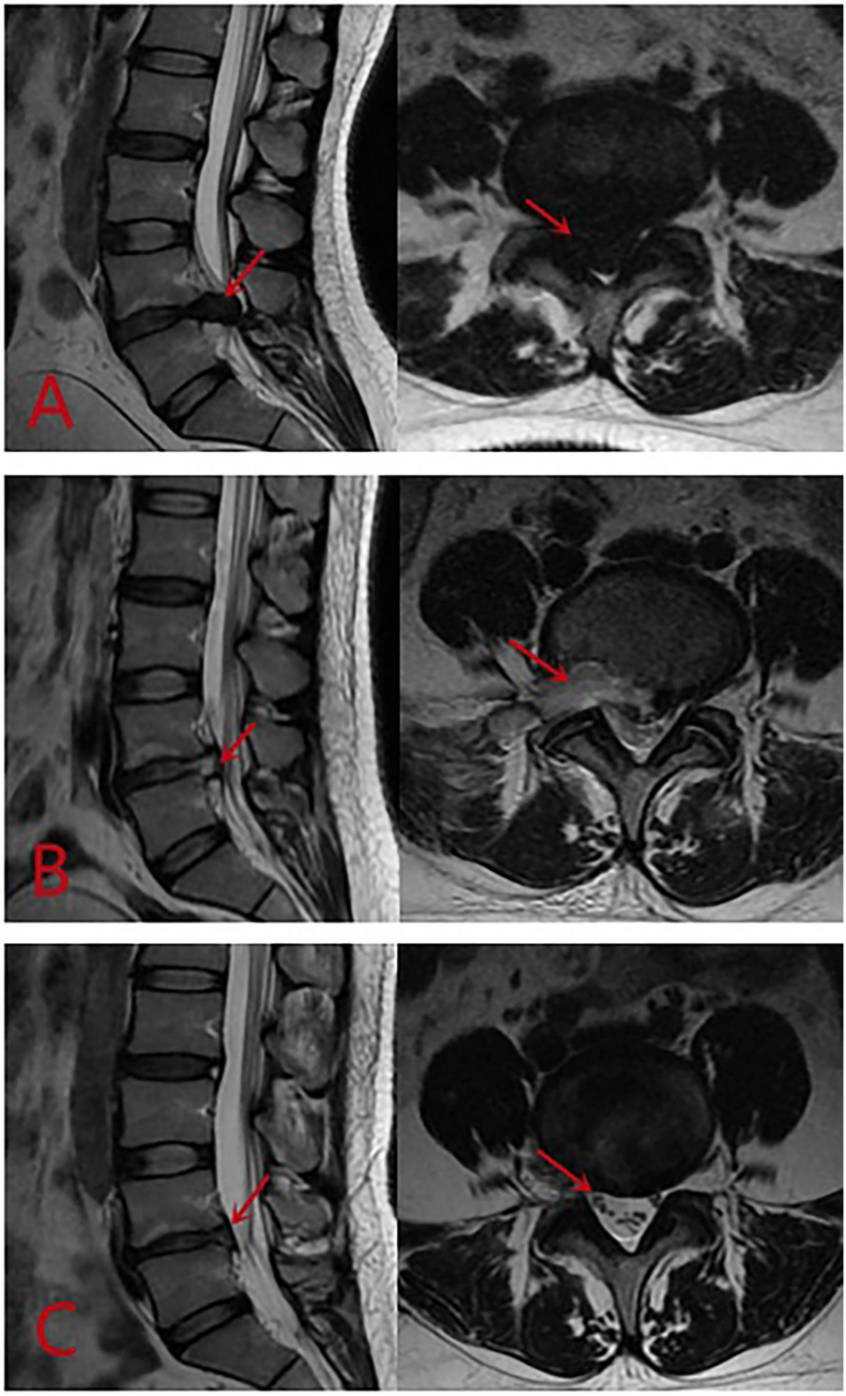
Preoperative MRI T2-weighted image showing a large central L5-S1 disc herniation causing severe compression of the cauda equina **(A)**, which was completely removed on the first postoperative day **(B)**, and the restoration of spinal canal volume to normal at 3 months after non-pressurized PETD surgery **(C)**.

All patients successfully underwent surgery, and the average follow-up time was 19.6 ± 4.2 months (range 13–28 months). Postoperative MRI examination in the PETD group showed adequate decompression of the spinal canal with relief of neural compression (Figure 3), while in the TLIF group, the screw and fusion device positions were satisfactory. The mean surgical duration was 70.4 ± 22.7 min (range 40–120 min) for the PETD group and 148.9 ± 29.2 min (range100–200 min) for the TLIF group. The difference in surgical duration between the two groups was statistically significant (*P* < 0.001), with the PETD group showing shorter surgical duration. The mean length of hospital stay was 6.8 ± 2.0 days (range 3–11 days) for the PETD group and 7.1 ± 2.4 days (range 4–14 days) for the TLIF group, with no significant difference observed between the two groups (*P* = 0.654).

**Figure 4:**
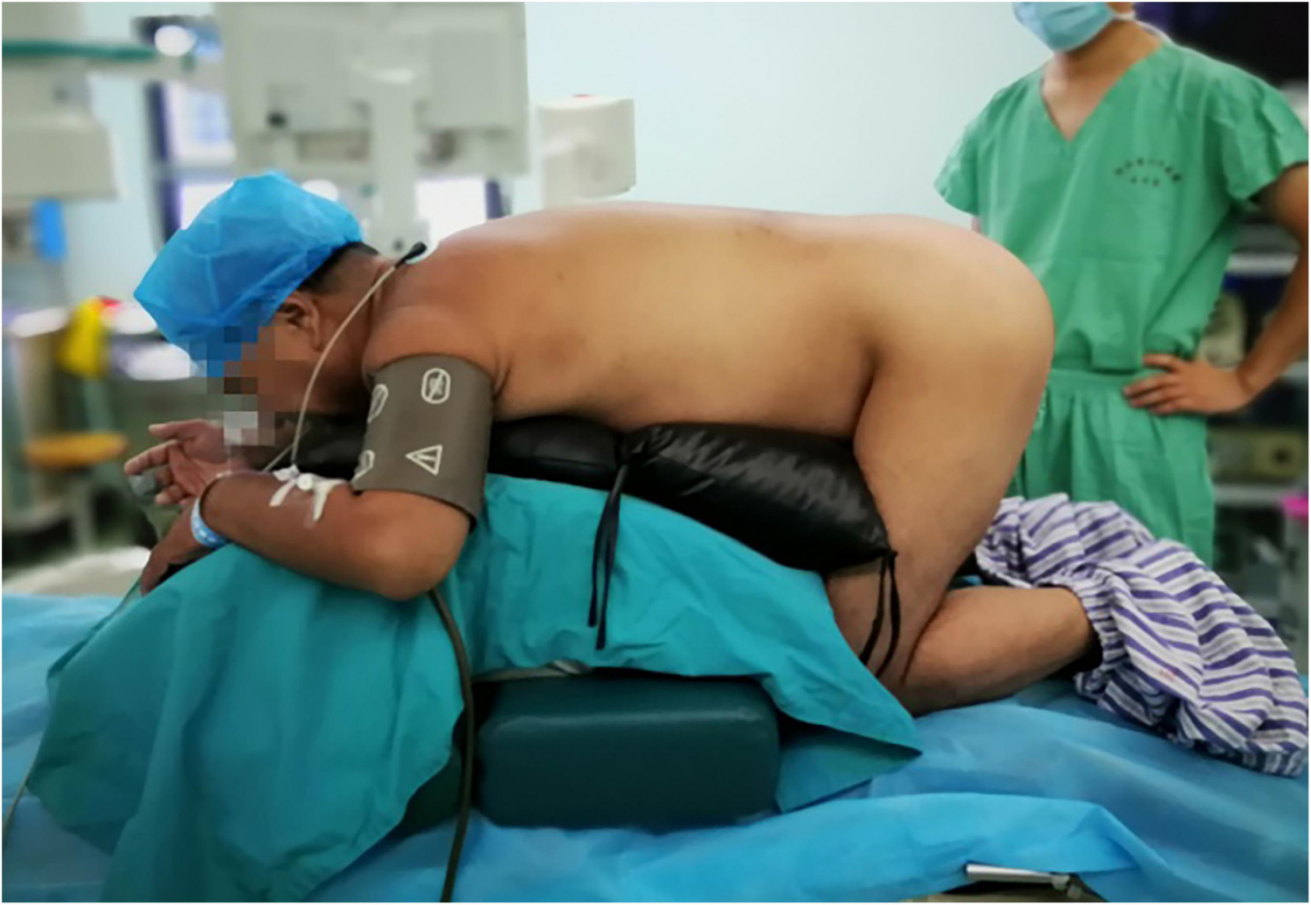
Intraoperative conversion to a kneeling position due to patient intolerance of prone positioning during non-pressurized PETD surgery. Sterility was maintained with adjusted draping.

**Figure 5:**
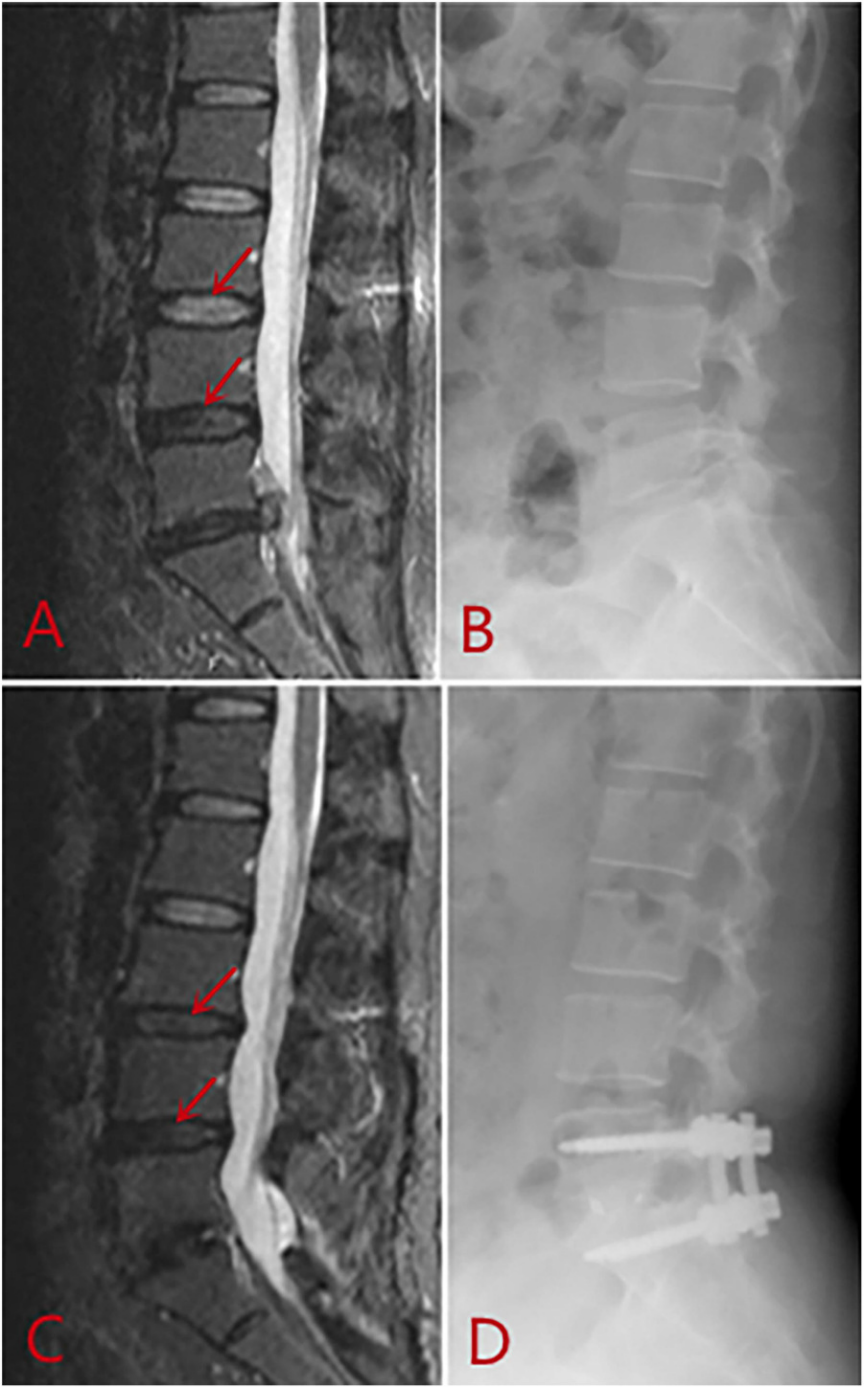
Compared with before surgery **(A,B)**, adjacent segment degeneration (red arrows) observed at the final follow-up 25 months after TLIF surgery **(C,D)**.

One case (1/23, 4.34%) of the PETD group could not maintain the prone position due to intraoperative pain, and the surgery was completed in the kneeling position (Figure 4). Neck pain occurred during the removal of the ligamentum flavum in two cases (2/23, 8.69%) of the PETD group, but the symptoms disappeared after the withdrawal of the endoscope. One case (1/23, 4.34%) experienced recurrent disc herniation with radiating leg pain but without CES symptoms five weeks after non-pressurized PETD, and symptoms were relieved after undergoing non-pressurized PETD again. For the TLIF group, adjacent segment degeneration was observed in one case (1/28, 3.57%) at the final follow-up (Figure 5), with mild symptoms of low back pain. There were no cases of severe complications such as nerve injury, epidural hematoma, or wound infection in either group.

Both PETD and TLIF groups showed significant improvement in low back pain VAS, leg pain VAS, JOA score, ODI score, and TCS score at the final follow-up compared to preoperative values (all *P* < 0.001). The PETD group showed better outcomes in terms of low back pain VAS (*P* = 0.011) and ODI score (*P* = 0.027) at discharge compared to the TLIF group. There were no significant differences in low back pain VAS, leg pain VAS, JOA score, ODI score, and TCS score between the two groups at preoperative and final follow-up assessments (all *P* > 0.05) (Table 3).

**Table 3:** Comparison of efficacy between the two groups at preoperative, discharge, and final follow-up.

Indicator	PETD group	TLIF group	Statistic	*P* value
Low Back Pain VAS*				
Preoperative	4 (3,6)	4 (3,6)	0.52	0.603
Discharge	2 (1,3)	3 (2,4)	2.55	0.011
Final Follow-up	2 (1,3)	2 (1,3)	1.23	0.220
*P* value**	<0.001	<0.001		
Leg Pain VAS*				
Preoperative	7 (5,8)	7 (5,8)	1.39	0.164
Discharge	2 (1,3)	2 (1,3)	0.09	0.930
Final Follow-up	2 (1,2)	1.5 (1,2)	0.24	0.814
* P* value**	<0.001	<0.001		
JOA*				
Preoperative	4 (3,8)	5 (3,7)	0.50	0.620
Discharge	19 (17,25)	19 (13,21)	1.55	0.121
Final Follow-up	23 (20,27)	23 (21,25)	0.19	0.849
* P* value**	<0.001	<0.001		
ODI*				
Preoperative	48 (41,68)	59 (52,67)	1.23	0.218
Discharge	38 (30,46)	41 (37,50)	2.21	0.027
Final Follow-up	25 (21,29)	21 (17,26)	1.43	0.152
* P* value**	<0.001	<0.001		
TCS*				
Preoperative	5 (5,7)	5 (4,7)	0.95	0.345
Discharge	6 (5,8)	6 (5,7)	1.26	0.208
Final Follow-up	7 (6,8)	7.5 (6.25,8)	0.13	0.899
* P* value**	<0.001	<0.001		

Data are presented as median (interquartile range).

* VAS, visual analog scale; JOA, Japanese orthopaedic association score; ODI, oswestry disability index; TCS, the cauda scale.

** The final follow-up and preoperative values of each group were compared.

Post hoc power analysis was performed, and equivalence tests for the difference between two means were selected due to the similar clinical outcomes of the two groups at the final follow-up. Among the clinical assessment variables, ODI score was used because of its difference between the two groups was largest, and the minimal clinically important difference of ODI was 12.8 according to the literature reports (22). Equivalence limits were set as −12.8 to 12.8, actual difference was calculated as 4, and standard deviation was calculated as 11.2. Based on these parameters, sample size as 23 cases in PETD group and 28 cases in TLIF group provided 86.60% power.

## 5 Discussion

CES is a rare clinical syndrome in spinal surgery, which often has a poor prognosis and significant impact on daily lives of patients, making it a challenging condition for spinal surgeons (1). The key point of surgical treatment is to relieve compression of the cauda equina and provide good space and blood supply for its self-repair. However, even with adequate decompression, the nerve function of the cauda equina often cannot get complete recovery because of irreversible damage caused by severe compression to the cauda equina (5, 6). Early decompression surgery is one of the key factors affecting the prognosis of patients, so surgery was performed within 48 h of admission in this study.

CES also sometimes occurs as a complication after traditional PETD or traditional open decompression and intervertebral disc removal surgery, with an incidence of approximately 5 cases per 1,000 procedures (2). In addition to direct damage to the cauda equina, compression and excessive traction of the cauda equina by surgical instruments are also important reasons. Therefore, it is worth noting to avoid iatrogenic traction and compression of cauda equina during surgery. Especially for patients with CES, intraoperative interference with the cauda equina is fraught with risks because the cauda equina remains in an extremely vulnerable state.

The cauda equina was sufficiently decompressed by traditional posterior spinal canal decompression and intervertebral disc removal surgery, which provided an open field of view and a large exploration area. However, during the procedure, the cauda equina may undergo a process of traction and pressure before decompression, which is not conducive to the recovery of cauda equina function. Similarly, during the traditional PETD procedure, the working channel is placed directly into the target site of the protruding intervertebral disc after puncture and facet formation, which increases pressure within the spinal canal and causes some degree of pushing and traction on the cauda equina, resulting in increased pressure before decompression. In this study, the use of non-pressurized PETD technique avoids compression, traction, and increased intraspinal pressure by gradually advancing the working channel into the intervertebral foramen.

One of the advantages of PETD operation is shorter surgical duration compared with TLIF operation (23), which was also observed in this study. An important reason is the difference in the complexity of the operation. Non-pressurized PETD only underwent decompression of the spinal canal and exploration of nerve roots, and the surgical duration was 70.4 ± 22.7 min, which was similar to that of traditional PETD. In contrast, traditional TLIF requires additional steps such as interbody fusion and internal fixation. Another advantage of PETD operation is shorter hospital stay compared with TLIF operation (23). However, the hospital stay of the two surgical methods was similar, which was about seven days in this study. This is because compared with patients with simple LDH, patients with CES caused by LDH require additional time for neurological function observation, urinary catheter care and bladder training after surgery, not only depending on the surgical situation.

Patients achieved significant improvement in VAS of low back Pain, VAS of leg pain, JOA, ODI, and TCS after non-pressurized PETD surgery, which was comparable to TLIF, consistent with previous studies (10, 23, 24). Li et al. (24) reported that in 16 patients with CES who underwent PETD, including transforaminal and interlaminar approaches, the VAS for leg pain decreased from 7.67 ± 1.23 before surgery to 1.71 ± 0.53 after surgery, with a good rate of 81.2%. Dave et al. (10) reported that the long-term efficacy of simple decompression surgery appeared to be comparable to fusion surgery in 64 patients with cauda equina syndrome, but the satisfaction rate of patients undergoing fusion surgery was higher. The TCS score was significantly improved after operation in this study, indicating a good recovery of cauda equina function. However, there are currently no reports of other surgical methods using TCS, nor quantitative comparisons of cauda equina function.

One patient experienced recurrent disc herniation after non-pressurized PETD surgery, which may be due to less preservation of the posterior longitudinal ligament and annulus fibrosus to achieve complete decompression of the cauda equina during surgery. Two patients experienced neck pain during non-pressurized PETD surgery, which was thought to be due to increased pressure on the dura mater from continuous hydrostatic irrigation, which often occurs when decompression is almost complete or cerebrospinal fluid leaks. Joh et al. (25) found that the probability of neck pain increased significantly when the extrathecal pressure in the cervical region exceeded 37 mmHg, which was related to the volume and speed of water irrigation. There are currently no reports of cauda equina nerve damage caused by continuous water flushing during surgery, but continuous water flushing in endoscopic surgery does increase pressure on the cauda equina in theory. Therefore, in endoscopic surgery for CES, high-pressure, high-flow, and long-term water flushing should be avoided.

The key points of non-pressurized PETD technique are as follows. (1) Unlike traditional PETD, the non-pressurized technique for foraminoplasty is performed under endoscopy using drills and Kerrison rongeurs, which can effectively avoid the increase of iatrogenic intraspinal pressure caused by compression of facet blocks under fluoroscopy or visual inspection, resulting in pushing of nerves. (2) While traditional TLIF requires partial traction of the nerve roots and dural sac towards the midline to expose central disc herniation, the non-pressurized PETD technique enters the spinal canal from the intervertebral foramen at a more parallel angle to the ventral side of the spinal canal without the need to pull the nerve roots or dural sac. This is one of the reasons why we recommend avoiding PETD via the interlaminar approach in the treatment of CES associated with LDH. (3) In order to avoid excessive operation in the spinal canal, this technique requires that herniated disc entering the spinal canal should be restored to the intervertebral space before being removed. (4) Complete nerve decompression is the key to the recovery of cauda equina nerve function. Therefore, resection of the posterior longitudinal ligament and annulus fibrosus is necessary, once they are determined to compress the cauda equina nerve during intraoperative exploration, although such removal may increase the possibility of recurrence.

This study has some limitations. First, this was a retrospective study with a relatively small number of cases. Second, during medical record retrieval, there was no available case data on traditional PETD surgery for LDH secondary to CES. Therefore, the advantages of non-pressurized technology were described based on the data reported in the previous literature on traditional PETD. Third, the evaluation lacked assessor blinding and objective urodynamic validation. Finally, the follow-up time was 19.6 ± 4.2 months, while adequate for pain scores, may be too short to fully assess permanent CES functional deficits.

## 6 Conclusion

The key points of non-pressurized PETD technique in the treatment of LDH complicated by CES lie in the gradual decompression of the spinal canal under endoscopy from the intervertebral foramen. In this preliminary retrospective study, the non-pressurized PETD technique was associated with similar medium-term outcomes and a faster short-term recovery compared to TLIF. These findings require validation in larger, prospective, randomized controlled trials to establish comparative efficacy.

## Data Availability

The raw data supporting the conclusions of this article will be made available by the authors, without undue reservation.
